# Evaluation of Small Airways Dysfunction With Dupilumab Using Airway Oscillometry in Uncontrolled Severe Asthma

**DOI:** 10.1111/cea.70002

**Published:** 2025-01-30

**Authors:** Kirsten E. Stewart, Chris RuiWen Kuo, Rory Chan, Brian J. Lipworth

**Affiliations:** ^1^ Scottish Centre for Respiratory Research, School of Medicine University of Dundee, Ninewells Hospital Dundee UK

**Keywords:** asthma, biologics, dupilumab, oscillometry, small airways dysfunction

1

**Summary**

- Improvements in peripheral resistance and compliance occurred with dupilumab in patients with severe asthma.
- Dupilumab also conferred improvements in forced mid expiratory flow and asthma control.

To the Editor,

1

Dupilumab acts on IL‐4 receptor alpha inhibiting IL‐4 and IL‐13 signalling which in turn attenuates type 2 inflammation (T2I) in patients with asthma. The presence of small airway dysfunction (SAD) may be assessed using airway oscillometry (AO) which is performed during normal tidal breathing. AO may exhibit a high degree of sensitivity in detecting SAD in asthma, which can be evaluated using either peripheral lung resistance as the heterogeneity in resistance between 5 Hz and 20 Hz (R5‐R20) or peripheral lung compliance as the area under the reactance curve (AX).

The presence of SAD as increased R5‐R20 in asthmatic patients with an FEV_1_ > 80% predicted is associated with significantly greater use of oral corticosteroids and salbutamol [1]. Moreover, SAD as altered R5‐R20 and AX, but not as FEV_1_, is closely related to T2I biomarkers [2]. A real‐life retrospective study in patients with uncontrolled severe asthma receiving dupilumab revealed improved SAD detected through R5‐R20 and AX [3]. The objective of the present study was to prospectively assess the impact of 12 weeks of treatment with dupilumab on SAD in patients with type 2 high (T2H) poorly controlled severe asthma.

This was a phase 4 single‐arm proof of concept clinical trial with a single‐centre, open‐labelled design which included adults aged 18–75 years with T2H poorly controlled severe asthma despite taking ICS/LABA with or without other second line controllers. Eligible patients entered a 4‐week run‐in period with standardisation of ICS/LABA as maintenance and reliever therapy (MART) 2–8 actuations per day using extra fine particle Fostair NEXThaler 100/6μg dry powder inhaler beclomethasone dipropionate/formoterol (BDP/FM). A treatment period of 12 weeks of dupilumab was then employed with an initial 600 mg loading dose followed by 300 mg 2 weekly doses in addition to BDP/FM MART. Participants were then followed for a subsequent 12‐week washout period without dupilumab to week 24. ERS and ATS guidelines were followed for spirometry (Micromedical, Chatham, UK) and airwave oscillometry (AO) (TremoFlo c100, Thorasys, Montreal, Canada) according to the manufacturer's instructions and following published guidance [4]. The study was approved by a local research ethics committee (21/WS/0151) and all participants gave written informed consent. The clinical trial was registered with ISRCTN:70810039. The presence of SAD at baseline was defined using values as follows: R5‐R20 ≥ 0.1 kPa/L/s, R5‐R20/R5 Ratio ≥ 19%, AX ≥ 1.0 kPa/L and FEF_25–75_ ≤ 60% predicted [5]. Repeated measures analysis of variance (RM‐ANOVA) was initially performed to compare results overall between baseline, week 12 and week 24. In the presence of a significant overall RM‐ANOVA, pairwise Students *t*‐tests were used to assess differences between baseline (week 0) versus post dupilumab (week 12), and between week 12 versus washout (week 24). A post hoc responder analysis was then performed to assess the proportion of patients who had changes from baseline at week 12 which exceeded respective minimal clinically important difference (MCID) values [6].

Twenty‐four participants completed 12 weeks of dupilumab, and 20 participants completed the washout week 24 due to loss of control in four patients. Mean (SEM) baseline values were age (years) 52 (3), FEV_1_ (% predicted) 82 (3), ICS dose (μg) 1300 (91), Total IgE (kU/L) 205 (43), blood eosinophils (cells/μl) 552 (54), FeNO (ppb) 50 (7) and ACQ‐5 2.65 (0.19), BMI 30 (1.2). Mean ICS dose as extra fine BDP equivalent prior to run‐in was 650μg (45)/day. There were significant differences for all outcomes at week 12 compared to baseline, while after washout at week 24 compared to week 12 only R5‐R20 and ACQ‐5 were significantly different (Table 1). The MART dose of extra fine particle BDP/FM was reduced by a mean of 1.7 puffs/day (95% CI: 0.7–2.7) *p* < 0.01, from a mean of 5.2 puffs/day at baseline (520 μg/day extra fine BDP equivalent dose) to 3.6 puffs/day (i.e., 360 μg/day extra fine BDP equivalent dose) at 12 weeks.

**TABLE 1:** Mean and 95% CI values at week 0 (baseline), week 12 (post dupilumab), week 24 (washout), and mean differences (95% CI) between week 12 versus week 0 and between week 24 versus week 12.

	Week 0 (baseline)	Week 12 (post dupi)	Week 24 (post washout)	Week 12 vs. week 0. mean diff (95% CI)	Week 24 vs. week 12. mean diff (95% CI)
R5 (kPa/)L/s	0.49 (0.41, 0.57)	0.41 (0.33, 0.48)	0.39 (0.28, 0.50)	−0.09 (−0.01, −0.17)*	−0.02 (−0.10, 0.06)
R5‐R20 (kPa/L/s)	0.125 (0.076, 0.174)	0.067 (0.032, 0.102)	0.139 (0.070, 0.209)	−0.058 (−0.094, −0.022)**	0.064 (0.002, 0.127)*
R5‐R20/R5 Ratio (%)	21.6 (14.6, 28.6)	13.2 (6.2, 20.2)	25.9 (10.4, 41.4)	−8.44 (−13.13, −3.75)**	11.69 (−3.36, 26.75)
AX (kPa/L)	2.59 (1.46, 3.71)	1.32 (0.65, 2.00)	2.07 (0.88, 3.27)	−1.26 (−2.18, −0.34)**	0.64 (−0.18, 1.47)
X5	−0.29 (−0.37, −0.20)	−0.17 (−0.22, −0.12)	−0.25 (−0.35, −0.15)	0.10 (0.05, 0.17)**	−0.07 (−0.16, 0.01)
F_res_ (Hz)	21.16 (17.85, 24.47)	17.68 (14.38, 20.97)	19.63 (15.83, 23.43)	−3.31 (−0.26, 3.62)**	1.68 (−0.26, 3.62)
FEF25‐75 (L/s)	1.47 (1.20, 1.74)	2.06 (1.53, 2.60)	1.82 (1.33, 2.30)	0.59 (0.23, 0.95)**	−0.20 (−0.50, 0.10)
ACQ‐5	2.65 (2.25, 3.05)	0.84 (0.44, 1.24)	2.17 (1.69, 2.66)	−1.81 (−2.26, −1.35)***	1.50 (0.94, 2.05)***

*Note:* R5, total lung resistance at 5 Hz, R5–R20; peripheral lung resistance as heterogeneity between 5 Hz and 20 Hz; AX, peripheral lung compliance as area under the reactance curve; X5, reactance at 5 Hz; F_res_, resonance frequency; FEF_25–75_, forced mid expiratory flow rate between 25% and 75% of forced vital capacity; ACQ‐5, 5‐item asthma control questionnaire. *p < 0.05, **p < 0.01, ***p < 0.001.

Responder analysis comparing week 12 versus baseline showed improvements exceeding MCID's in R5‐R20 ≥ 0.04 kPa/L/s occurring in *n* = 10/24 (42%), AX ≥ 0.39 kPa/L *n* = 12/24 (50%), FEF_25‐75_ ≥ 0.21 L/s *n* = 17/24 (71%), and ACQ‐5 ≥ 0.5 *n* = 23/24 (96%).

Those meeting the presence of defined SAD at baseline had the following mean (95% CI) differences at 12 weeks compared with baseline: *n* = 12/24 (50%) had R5–R20 ≥ 0.1 kPa/L/s and improved by −0.105 (−0.166, −0.438) *p* < 0.01; *n* = 12/24 (50%) had R5–R20/R5 ratio ≥ 19% and improved by −13.24 (−20.32, −6.16) *p* < 0.01; *n* = 16 (67%) had AX ≥ 1.0 kPa/L and improved by −1.86 (−3.17, −0.55) *p* < 0.01; and *n* = 22 (92%) had FEF_25–75_ ≤ 60% predicted and their FEF_25–75_ (L/s) improved by 0.57 (0.18, 0.97) *p* < 0.01.

The present study showed that SAD outcomes derived from AO and spirometry improved significantly with dupilumab despite evidence of ICS sparing with MART therapy. After washout, there was a noticeable trend towards worsening of SAD outcomes versus week 12 which was significant only for R5‐R20 and was accompanied by significant worsening of ACQ‐5 score. Whether this might indicate a time lag difference between subjective and objective outcomes is unclear, or perhaps due to type 2 error. IL‐4 and IL‐13 but not IL‐5 are highly expressed in human small airway smooth muscle [7], which in turn is likely to explain the observed improved improvements in AO in the present study. Effects of dupilumab on ciliary function and mucin expression are mediated by IL‐13 [8]. Svenningsen et al. demonstrated significant impacts with dupilumab versus placebo in terms of mucus plugging, remodelling, gas trapping and ventilation [9]. In particular, comparison with the AO data of Svenningsen et al. [9] using the same device in 13 patients receiving dupilumab for 16 weeks showed mean improvements versus placebo amounting to −0.063 kPa/L/s for R5–R19 and 1.43 kPa/L in for AX, which are similar to the present findings.

We appreciate the limitations of our study including our sample size of participants meeting SAD defined criteria at baseline being small. However, this study was a prospective analysis in a well‐defined cohort of severe asthmatics in terms of T2H phenotype and disease severity. Notably the MCID cut points we used for our responder analysis were derived using impulse oscillometry which compared to airwave oscillometry is associated with lower values for AX along with higher values for R5‐R20.

## Author Contributions

K.S. data collection, writing, statistical analysis and review. C.K. trial design and submission, review. R.C. trial design and submission, review. B.L. trial design, data interpretation and analysis, writing.

## Conflicts of Interest

Ms. Stewart reports no conflicts of interest. Dr. Kuo reports personal fees from AstraZeneca, personal fees from Chiesi and non‐financial support from GSK outside the submitted work. Dr. Chan reports personal fees (talks) and support attending ERS from AstraZeneca, personal fees (consulting) from Vitalograph and personal fees (talks) from Thorasys. Dr. Lipworth reports non‐financial support (equipment) from GSK; grants, personal fees (consulting, talks and advisory board), other support (attending ATS and ERS) from AstraZeneca; personal fees (talks and consulting) from Sanofi and Regeneron, personal fees (consulting, talks and advisory board) from Circassia; grants, personal fees (consulting, talks, advisory board), other support (attending ERS) from Teva; personal fees (talks and consulting), grants and other support (attending ERS and BTS) from Chiesi; personal fees (consulting) from Lupin, personal fees (consulting) from Glenmark; personal fees (consulting) from Dr. Reddy; personal fees (consulting) from Sandoz; grants, personal fees (consulting, talks, advisory board), other support (attending BTS) from Boehringer Ingelheim; grants and personal fees (advisory board and talks) from Mylan outside of the submitted work; and the son of BJL is presently an employee of AstraZeneca.

## Data Availability

The data that support the findings of this study are available from the corresponding author upon reasonable request.
